# Humanistic health of the physical living environment: The equity of property inheritance in China

**DOI:** 10.3389/fpubh.2023.1147107

**Published:** 2023-04-11

**Authors:** Mengrong Shu, Jinxin Li, Yuhan Wu, Kaida Chen, Shuhui Ding

**Affiliations:** ^1^Fuzhou Technology and Business University, Fuzhou, China; ^2^Suzhou Polytechnic Institute of Agriculture, Humanity and Social and Development College of Nanjing Agricultural University, Suzhou, China; ^3^College of Landscape Architecture and Art, Fujian Agriculture and Forestry University, Fuzhou, China; ^4^Department of Urban Planning, National Cheng Kung University, Tainan, Taiwan

**Keywords:** cultural heritage, traditional residence, family culture, property inheritance, spatial syntax, architectural physics, humanistic health

## Abstract

Equity in the inheritance culture of family property is an important component of humanistic health in modern living environments. The inheritance of property under Chinese traditional family culture is the material basis for the continuation of family and clan. This study demonstrates the equity component embedded in traditional family inheritance culture and further studies of the healthy human settlements environment. Based on the theory of “equal share for all sons” in ancient China and the “equity” and “justice” that are of modern significance, this paper analyzes the family division culture of individual traditional housing and the corresponding impact indices of family division equity. Taking Renhe Village, a typical local residential building of the middle and late Qing Dynasty as the research object, this study built a spatial syntax data model and 3D simulation technology for the simulation analysis of space and climate. The results show that Renhe Village meets the requirements of the equity evaluation system of housing property rights distribution in terms of the natural unit indicator (quantity, lighting, ventilation) and the overall spatial indicator (privacy, centrality, convenience). In other words, equity does not mean an absolute average share, but an equity culture formed after six evaluation indices under the subdivision of two indicators are balanced. Based on the above, an equity system model of housing property rights distribution was established, and the weight of the ancients' attention to the housing distribution standard was explored. It is further found that the ancients attached more importance to light among the natural unit indicators, and attached the most importance to centrality in the overall spatial indicators. These findings provide new ideas for understanding the equity of property inheritance under Chinese traditional family culture. They also provide quantifiable criteria for the distribution of modern rural housing and social security housing, and ultimately provide a reference for the humanistic public health of the modern living environment.

## 1. Introduction

### 1.1. Background

Humanistic health is crucial to the physical living environment. One of the important issues in humanistic health is residents' pursuit of equity, including the equity of family property inheritance. Family culture is at the core of Chinese traditional culture, and the inheritance of family property is an important way of maintaining family continuity. Chinese family culture advocates the large family model of “many generations living together” (1–3). To avoid family friction and conflict caused by the existence of multiple interest centers in large families, most of them choose to divide up the family property after experiencing the development from small to large. In this way, family property is divided up equally according to the number of small families, which means that property ownership is redefined. Family division realizes the succession of new and old families through the transfer of property, power, and responsibility to ensure the reproduction of families and the continuity of Chinese families. As the concept of the house is male-oriented, the first condition for the existence of a house is that the ancestor must have several sons, and the daughters have no right of inheritance. The property is divided according to the number of sons (4). The formal division of family property is an important sign of family division, after which each son's family forms its own independent branches, inheriting the blood and property of the father equally and promoting the continuous development of the family.

The property inheritance system under Chinese family culture is characterized by primogeniture and “equal share for all sons”. In the major civilizations of the world, the main form of family continuity is primogeniture. Parents pass on their property to the eldest son, and the other children make a living in their own way and the family maintains an overall continuity. Therefore, the system is not “family division”. Primogeniture also existed in certain periods in Chinese society. During the Shang and Zhou dynasties, under the feudal system, primogeniture was the main form of inheritance, and the ruling power, the state, the people, and property were all included in the scope of inheritance. Therefore, primogeniture is more about the noble titles and the succession to the throne of the emperor (5). With the decline of the enfeoffment system and the hereditary system, the scope of inheritance of the legitimate eldest son in ancient China became narrower, and the common people generally gave an equal share to all the sons (6). Since the Qin and Han Dynasties, the important feature of the property inheritance system of the ancient Chinese family is that all the descendants inherit equally, no matter how much land and family property they owned (7). Due to the long-term implementation of the property inheritance system of an “equal share for all sons” and the stability of the farming environment, population growth in traditional Chinese society was rapid (8). The “equal share for all sons” system reverses the balance of the land concentration trend, playing a regulatory role in the excessive concentration of land (9). This situation shows that the way of household division represented by the “equal share for all sons” is a production and operation mode that is compatible with the level of social and economic development in the feudal era, and its emergence and popularization was a historical necessity (10). The equity of the “equal share for all sons” system can be illustrated by an interesting story. According to *The Sequel to the Biography of Yan Lun of Wu*,^1^ written by Wu Jun in Liang State of the Southern Dynasty, in the Han Dynasty, three brothers, Tian Zhen, Tian Qing, and Tian Guang, divided the family property and even divided the bauhinia tree in the courtyard. They cut the tree into three sections and gave one section to each family to show equity (11). This shows that, in traditional Chinese society, people were meticulous about the equity of family division and that equity was an important cornerstone of humanistic health and social stability.

The distribution of real estate property rights under the “equal share for all sons” system is not purely egalitarian. The overall balance should also be considered. This includes the equal distribution principles of “reducing more to replenish less”^2^ and “intersection and interpenetration”.^3^ Lao Tzu^4^ pointed out from the perspective of “Way of Heaven”^5^ that “reducing more to replenish less” (see text footnote ^2^) is the embodiment of social equity thought (12). In the distribution of real estate property, it refers to reducing the amount of real estate property to those who already have sufficient property and giving more to those who do not already own much real estate, thereby achieving balance and equity. Meanwhile, to maintain the stability of family settlements, a distribution strategy of “intersection and interpenetration” would be adopted in the distribution of the real estate to family members to promote interaction among the various household branches^6^ and make them look out for one another while ensuring that the number of rooms and the related factors are fairly distributed, thereby ensuring equity within the family. In practice, after family division, small families become independent production and consumption units. However, in daily production and life, each house branch still maintains close contact with the others in a state of “coexistence in separation”^7^ (2, 13). Therefore, it is very common for them to have a shared entity after the family property division. This is manifested in the form of the mutual intersection and interconnection of land ownership (14). In summary, this “intersection and interpenetration” (see text footnote ^3^) and the Taoist idea of “reducing more to replenish less” together constitute the equity of inheritance and the division of real estate property. It ensures the principle of equality, fairness, and justice in the process of family property division, and it constitutes a means of resource allocation under the system of an equal share for all sons, thereby forming China's traditional and unique family culture.

“Family” space is an important carrier of family culture inheritance. It solidifies and embodies certain social norms and cultural customs (15). Space syntax provides a new method for exploring the deep cultural meaning of home space. Space syntax is a theory and method of studying the relationship between space organization and human society by quantifying the structure of human settlements, including buildings, settlements, cities, and even landscapes (16). The social and cultural logics carried by home space reflect the spatial and temporal attributes of home space, and the concentrated embodiment of the relationship between people and society. The application of space syntax to the family is called “space archaeology.” It defines rules in its spatial fabric from a large number of cross-cultural architectural plan studies and then analyzes whether these rules are systematically related to a specific cultural logic. On this basis, it infers various supports of home space for family life and organization methods and reveals the underlying social and cultural logic (17). Finding idiosyncratic genotypes has always been the focus of space syntax research on home spaces (18). In the early days of the establishment of space syntax, Hillier et al. analyzed the fabric characteristics of home space in rural areas of southern France using spatial connectivity, the RRA value, and the difference value. They summarized the genotypes of home space in southern France from the perspectives of social culture, behavior mode, and daily family activities (19). Orhun found that there are two different spatial models in Turkey: kitchen integration and living room integration. The difference in the syntactic dimensions of different models reflects the difference in the genotypes of houses. The existence and distribution of these genotypes of houses are closely related to the migration and integration of nomadic people in Turkey (20). Tao Wei and Ding Chuanbiao discussed whether the fabric and characteristics laws of different spaces correspond to certain cultural concepts and lifestyles. Their innovative research method could help reveal the deep cultural genotypes of home space (17). Ke et al. conducted a case study on the spatial form of traditional residential space in southern Sichuan by using spatial syntax to demonstrate that the unique environment of traditional residential space in southern Sichuan has created a complex and varied spatial form and a diversified mixture of human residence culture (21). In other words, in the home space, the occupation and use of the internal family spaces are closely related to the social culture of specific regions and nations.

Home space has been deconstructed and reconstructed in different social periods. The exploration of the temporal attributes of space forms in cultural logic involves tracing the history of the space syntax of the home space and the transfer of culture (22). Bendik Ma-num selected 150 sample apartments constructed at different times after 1930 in Oslo, the capital of Norway, and compared the differences in the use of family internal space under different areas and spatial fabrics. The internal family space still has a very clear functional partition, and the integration of each functional space was significantly different. The research revealed that the diverse needs of people's lifestyles and family activities have not been satisfied in contemporary housing space design (23). Mustafa et al. applied the theory of spatial grammar in testing the spatial form of two types of housing layouts (traditional and modern) in Erbil city and detected the privacy level in their configuration through analysis and comparison (24). Yi et al. adopted space syntax to make an in-depth analysis of the process and logic of the practice of housing space at different stages of tourism development and found that the meaning of housing space is closely related to village community space and to even wider space. Housing space has completely exceeded the basic material category as a house, and its meaning generation depends on ethnic groups or villages in which individuals are closely connected. The construction of housing space meaning is multi-scale (25). Al-Mohannadi et al. used space syntax to conduct a comparative study of the space forms of traditional and contemporary Qatari houses, thereby promoting the development of traditional buildings based on social sustainability and urban renewal of the architectural environment (26). The above research shows that analyzing the evolution of spatial form and fabric in the process of housing evolution and exploring the evolution of the concept of family in the process of housing transformation is conducive to observing changes in society at the micro-scale. In summary, the application of space syntax to home spaces provides a new perspective for exploring the relationship between people and their living spaces.

The construction of a family space has a high degree of harmony with the natural environment, correspondingly generating many adaptive design methods and construction experiences aimed at adjusting the living environment. All buildings go through a process of continuous change and development. A successful building should promote this development through adaptability (27). Adaptive design is an active design method (28). Most buildings are still designed and built for specific purposes, with little consideration given to changing climate conditions or the future needs of residents (29). One of the challenges facing architectural development is the possibility of reconfiguring new space according to environmental changes and users' needs (30). Among many environmental factors, the light environment is one of the climatic conditions that can directly affect the building layout. El Zafarany et al. believed that the important factors affecting the lighting of a building include the internal and external light environment of the building, as well as the characteristics of the building itself (31), among which the external light environment is the basis for lighting analysis. In 1942, Moon took the lead in proposing a full overcast sky brightness distribution model based on a trigonometric function relationship (32). Subsequently, the International Commission on Illumination determined 15 sky-type models of relative brightness distribution based on the brightness data that were collected by scanning several places. Zhijia et al. studied the indoor light environment of a typical traditional residential building in Huizhou, China, from the comfort perspective, over a one-year on-site test and continuous monitoring of multiple indoor environmental parameters of a typical traditional residential building (33). Natural ventilation has always played an important role in residential buildings. The basic influencing factors of building ventilation include building orientation, natural wind, and building structure (34), among which natural wind includes seasonal wind and natural ventilation (35–37). Kleiven believed that natural ventilation mainly affects the layout and organization of external walls, roofs, and internal spaces (38). Gado selected the new Al-Minya urban buildings in Egypt as a case study to analyze the effectiveness of a natural ventilation mode in actual buildings. Aydin et al. applied Turkish architectural elements to improve the ventilation of residential buildings, studying the function and applicability of different architectural elements (39). Spentzou et al. found an effective way to solve the problem of energy-saving renovation of residential buildings in Greece by implementing renovation strategies with computational fluid dynamics simulation (40). Regarding local traditional Chinese buildings, Jihong conducted research on the energy-saving technology of traditional residential buildings in Jiangsu and Zhejiang provinces and demonstrated that the building layout and structural form can reasonably organize air flow, which plays an important role in improving the indoor thermal environment (41). Hua investigated the ventilation status of traditional and modern residential buildings south of the Yangtze River and proposed a ventilation design strategy for residential buildings through numerical simulation (42). These studies have laid a foundation for the study of building ventilation and lighting analysis.

As mentioned above, home space contains the relationship between spatial form and social and cultural logic. It is also compatible with the natural environment. It is a microcosm of the human-land relationship. As a form of multi-scale and open home space (43), Chinese traditional residential buildings have a unique form of equity culture under the system of an equal share for all sons. They also represent the concept of the construction of residential buildings suitable for the natural environment, a valuable aspect of the heritage of Chinese traditional architectural culture.

### 1.2. Materials

#### 1.2.1. Target

The Zhuangzhai building located in Yongtai county, Fujian province, on the southeast coast of China, is a traditional residential building with an emphasis on residential and defensive functions. It originated in the Tang Dynasty and has developed over hundreds of years. It is also the epitome of Chinese traditional family culture and humanistic health architecture. According to the official statistics on the protection and development of ancient villages in Yongtai county, the total number of ancient historical Zhuangzhai buildings is estimated to be more than 2,000. Having experienced banditry and reform, 146 villages are preserved, including 98 villages that occupy an area of more than 1,000 sq m (44). In 2022, Yongtai Zhuangzhai was selected for inclusion in the World Monuments Watch list issued by the World Monuments Fund. Yongtai Zhuangzhai maintains the property inheritance system of Chinese family culture. It is a residential building established based on family and continued by blood relationships. It is the physical manifestation of regional architecture culture and local social life production (45).

**Figure 1:**
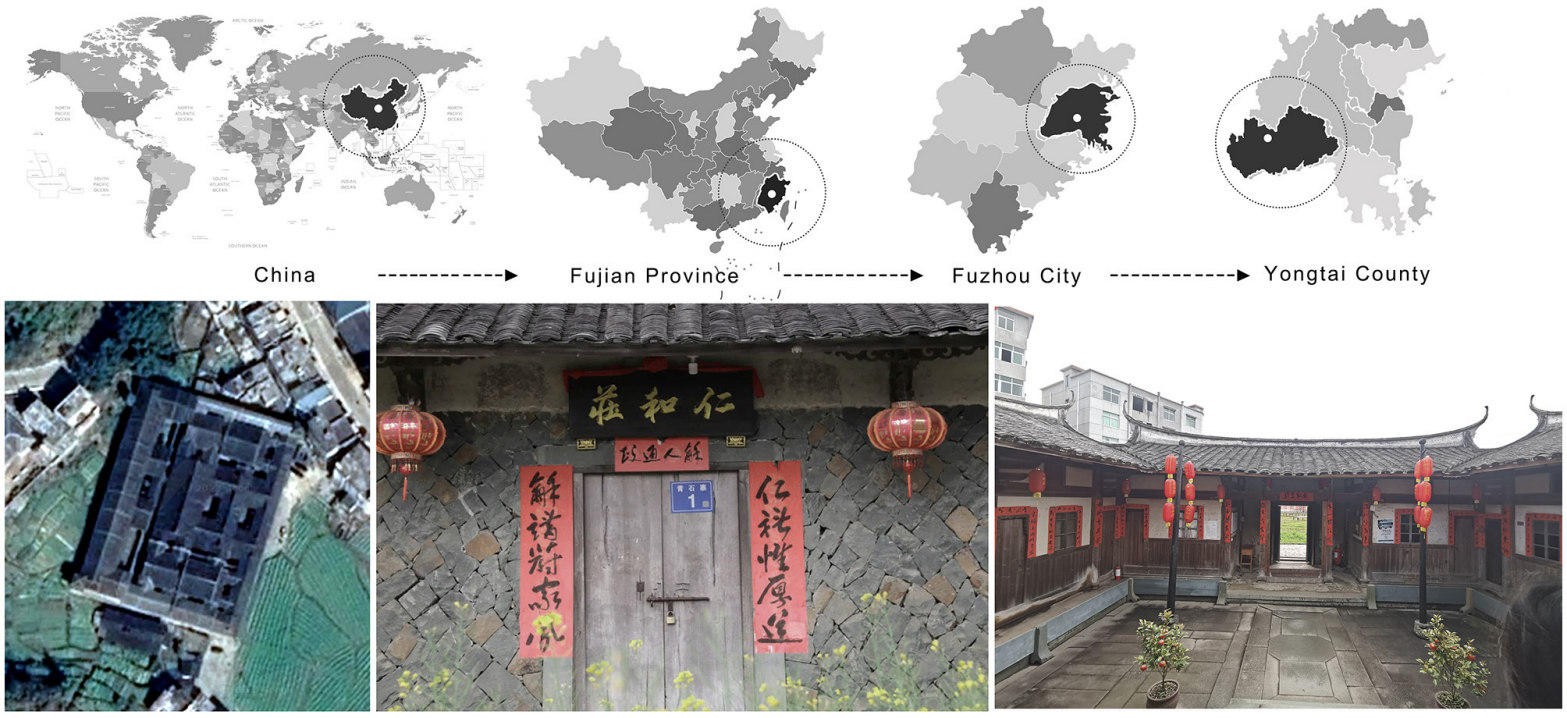
Particulars of Renhe Village.

Renhe Village (Figure 1) in Yongtai, which was built in the 10th year of Daoguang's reign in the Dynasty, is an important model of complete residential buildings under the “equal share for all sons” system. The village covers an area of more than 6,000 sq m, with a building area of 5,500 square meters, and it can accommodate hundreds of people. The plan of Renhe Village is a horizontal rectangle, arranged in the shape of a nine-square sudoku. The three axes are parallel, and the houses are located along the axes. The main gate is on the central axis, with three-entrance courtyards,^8^ each with a patio. There is good ventilation, lighting, and drainage. Renhe Village was jointly built by Zhang Xujie, Zhang Xuguang, and Zhang Xuyi, three brothers of the Zhang family. According to historical records, when the three Zhang brothers built Renhe Village, there were 12 male members in total in the three branches, and the brothers lived together. Later, when the descendants of the three brothers increased to 53, they decided to divide the family property, power, and responsibilities according to the system of “equal share for all sons”. This was done to avoid conflicts, which ensured the reproduction and continuity of the family. There is also a cultural meaning for the name Renhe Village. *The Book of Rites: Commentaries on the Classics* shows that “the closeness of superiors to subordinates is benevolence.”^9^ This commentary shows that “Ren” means benevolence (46), and “He” means “harmony”. “Ren” and “He,” benevolence and harmony, are the moral standards and spiritual character advocated by Confucianism. Therefore, the name “Renhe Village” expresses the owner's respect for and inheritance of Confucian culture. The owner expected his children to practice the concepts of benevolence and harmony.

#### 1.2.2. Research status

Renhe Village is a large village in central Fujian with a relatively complete form and structure. However, specific research on Renhe Village is limited. In his book *Fujian Zhuangzhai*, Jianjun and Zhuangzhai made a detailed introduction to the cultural value, spatial form, and decoration of more than 30 Zhuangzhai villages, including a detailed study of Renhe Village (47). Based on space syntax theory, Zhang Binghua summarized that the rule of the Zhuangzhai space plan layout, representing family internal ethics discipline, is to form different space connectivity and layout through space separation and opening and closing. The “body-space” coupling change strengthened the behavior discipline of ethical orders such as internal and external differences, the status of hosts and guests, and male superiority and female inferiority under specific scenarios (48). In the research area of equity in housing property rights, Cai Xuanhao revealed the architectural terminology system of Dacuo in East Fujian in the middle and late Qing Dynasty by interpreting the housing distribution documents of Renhe Village and Aijing Village. This research included aspects of the property rights distribution system (49). However, property inheritance under the traditional Chinese family culture also includes the distribution of living space in addition to farmland. Previous research paid little attention to the distribution of housing property rights, which, however, have a great impact on the living conditions of the next generation in a traditional society. The quantity and quality of housing are also important perspectives for the investigation of family living standards. The above review shows that existing research on the issue of equity of housing property rights in the family division culture of Renhe Village has the following limitations: (1) the interpretation of equity mainly focuses on the distribution methods of family division documents, a relatively limited area and (2) there is a lack of universal summaries in the interpretation of equity under inheritance culture. This makes it difficult to form an effective reference standard for future research on equity.

### 1.3. Value

Taking Renhe Village as the family house sample, this study analyzed the characteristics of housing distribution of traditional residential space and explains the equity of property inheritance under traditional Chinese family culture through in-depth data analysis. Based on the property inheritance system in traditional family culture, this study constructed a new equity evaluation system through the scientific analysis of actual data. Meanwhile, based on the reasonable source of internal equity, the equity model equation of housing property rights distribution was derived to verify the rationale of housing property rights distribution among brothers and to prove the equity of housing property rights distribution in traditional Chinese family culture. It is expected that this study will fill a research gap by carrying out research in this area, aimed at improving the comprehensive understanding of the value of traditional buildings, broadening the ideas for the equity design of contemporary architecture, and providing a reference for the humanistic health of the modern living environment.

This study consists of four chapters. The first chapter, the introduction, describes the background and object of the research and explains the major results and limitations of the existing research, clarifying the value of this paper. The second chapter is about the research methodology. It arranges the research framework, reveals the data sources, and makes a preliminary explanation of the research methodology by assuming the possibility of the research. The third chapter, the core of the study, confirms the equity evaluation system through a comprehensive analysis of the natural unit indicator (quantity, lighting, and ventilation) and the overall spatial indicator (privacy, centrality, and convenience) of the rooms. The fourth chapter is the conclusion. Based on the discussion and analysis, the equity model equation of property rights distribution is obtained. Finally, this study presents the results of the research and sets out the limitations of the research and directions for future research.

## 2. Methodology

### 2.1. Hypothesis

In the “equal share for all sons” system, equity is especially emphasized in the traditional Chinese housing division. This study assumed that the premise of equity is the objective. Therefore, a research framework was established to explore the constituents of equity. However, the number of houses distributed by the three brothers in the research sample of Renhe Village is different, and the location of these houses is complex. The achievement of equity in the distribution of housing property rights is a question to be addressed. Therefore, the following hypothesis is proposed: Equity is not determined by a single attribute, but by the complementarity of multidimensional attributes. Hence, it is of great importance to explore the proportion of the constituent indicators of equity.

### 2.2. Objectives

Based on systematically summarizing the connotation of property inheritance in Chinese family culture, this study takes Renhe Village as a sample to analyze the wisdom of equity contained in architecture. The research objectives are as follows: (1) The use of multidimensional research to enrich the horizontal dimension of the research on the equity of traditional family division culture, (2) the identification of the differences in the ancients' attention to the corresponding rooms of different residential buildings, and (3) the identification of the impact indicators of equity in the housing distribution of traditional residential buildings, and the establishment of equity indicators for the distribution of property rights of traditional residential buildings.

### 2.3. Framework

**Figure 2:**
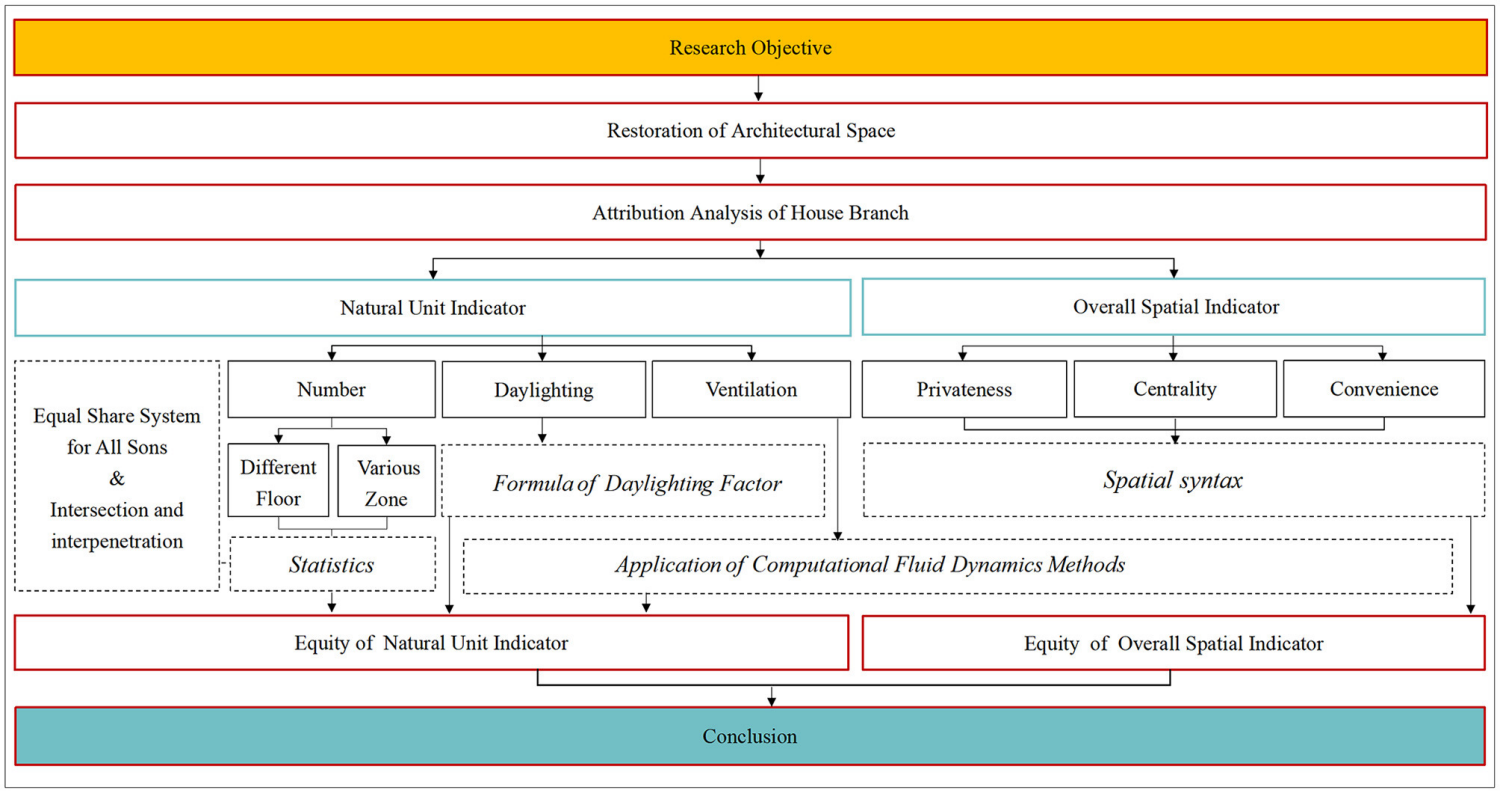
Framework of this study.

This study examined aspects of traditional Chinese culture, applying quantitative research to explore the equity of the distribution of traditional residential housing property rights. Figure 2 shows the main structure of the study. According to the arrangement of rooms, the structure is divided into two aspects. The first focuses on the natural unit indicator: the number of rooms in each area and the number of rooms with excellent lighting and ventilation quality. The second aspect is based on spatial topology and focuses on the overall spatial indicator and analyzes the characteristics of privacy, cultural centrality, and convenience of living in the overall location of the rooms.

The two indicators and six evaluation indices are described in this section. In terms of the natural unit indicator, this study found that there are as many as 316 rooms in Renhe Village. The number of rooms is important in the process of the property distribution of traditional residential buildings. The number of rooms and the quality of housing distribution determine the spatial form of the survival and reproduction of future generations. The physical attributes of lighting and ventilation are also important factors that determine the quality of rooms. In terms of the statistics of room locations, based on the quantity and location of rooms on each floor, this article studied the housing distribution strategy under traditional family culture, thereby analyzing the equity of the number of rooms in each household branch. This study also demonstrated the physical quality of room lighting and ventilation to determine the corresponding high-quality rooms. Finally, the equity of the natural unit indicator was proved through three evaluation indices: quantity, lighting, and ventilation.

In terms of the overall spatial indicator, the internal transportation of Renhe Village is complex. The internal living mode is combined with the lifestyle in the village, and the location of each room corresponds to different privacy, cultural centrality, and living convenience. Residential privacy (hereinafter referred to as “privacy”) is the basic demand of human beings in the life of each family unit. It is also a factor in the humanistic care of architecture for residents (50). The research on privacy is based on the average open horizon distance of the average housing unit of each household branch. This is taken as the privacy measurement standard. Cultural centrality (hereinafter referred to as “centrality”), meaning the ritual space at the core of architectural space, is selected as the midpoint. Traditional China is a ritualistic society. Therefore, there is a requirement for a cultural center for ritual activities in the residential space, a core ritual space for public affairs such as sacrifices, marriages, funerals, family meetings, and ceremonies. The main hall at Zheng Zuo^10^ is the geometric center of the plan pattern of the building complex and is the largest and highest-level building of all the single buildings (51). Therefore, the space distance between each room and the geometric center of the building is selected as the criterion of centrality. The main function of Renhe Village is living, and the convenience of transportation in the living process (hereinafter referred to as “convenience”) is one of the focuses of traditional residential housing distribution. The building entrance is an important node in the internal and external transportation link and the carrier of the transformation process between the internal and external spaces (52, 53). The stairs are not only a means of communication between the upper and lower floors but also form an entrance to the residential buildings (54). Therefore, in terms of convenience, the entrance is taken as the endpoint, and the average walking distance between each room and the entrance is counted as the criterion measuring convenience. It is worth noting that Renhe Village is a multi-story building. The distance residents go to reach the primary and secondary entrances is calculated as the evaluation criterion for the space on the first floor of the village. For the space above the first floor, the stairs are used as the entrance and exit to calculate the distance. Then, the distance from the position corresponding to the stairs on the first floor to the nearest entrance and exit is added. Therefore, the convenience of residents' internal and external transportation can be comprehensively analyzed. Finally, the three evaluation indices of privacy, cultural centrality, and convenience of living are taken as the influencing factors of the overall room indicator.

### 2.4. Data

**Figure 3:**
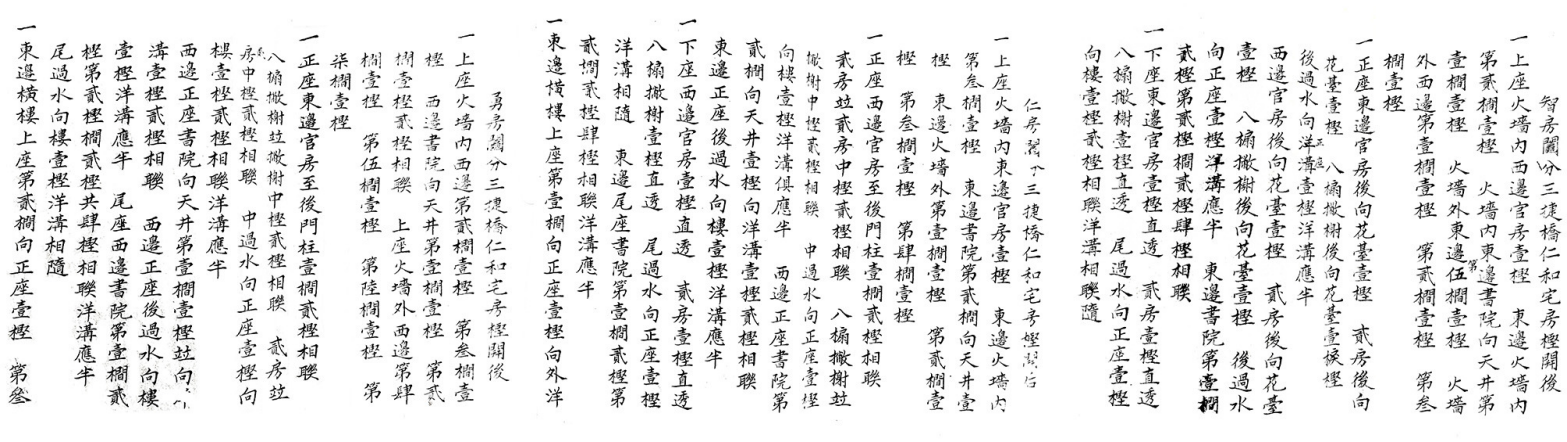
Jiu Book of Renhe Village.

**Figure 4:**
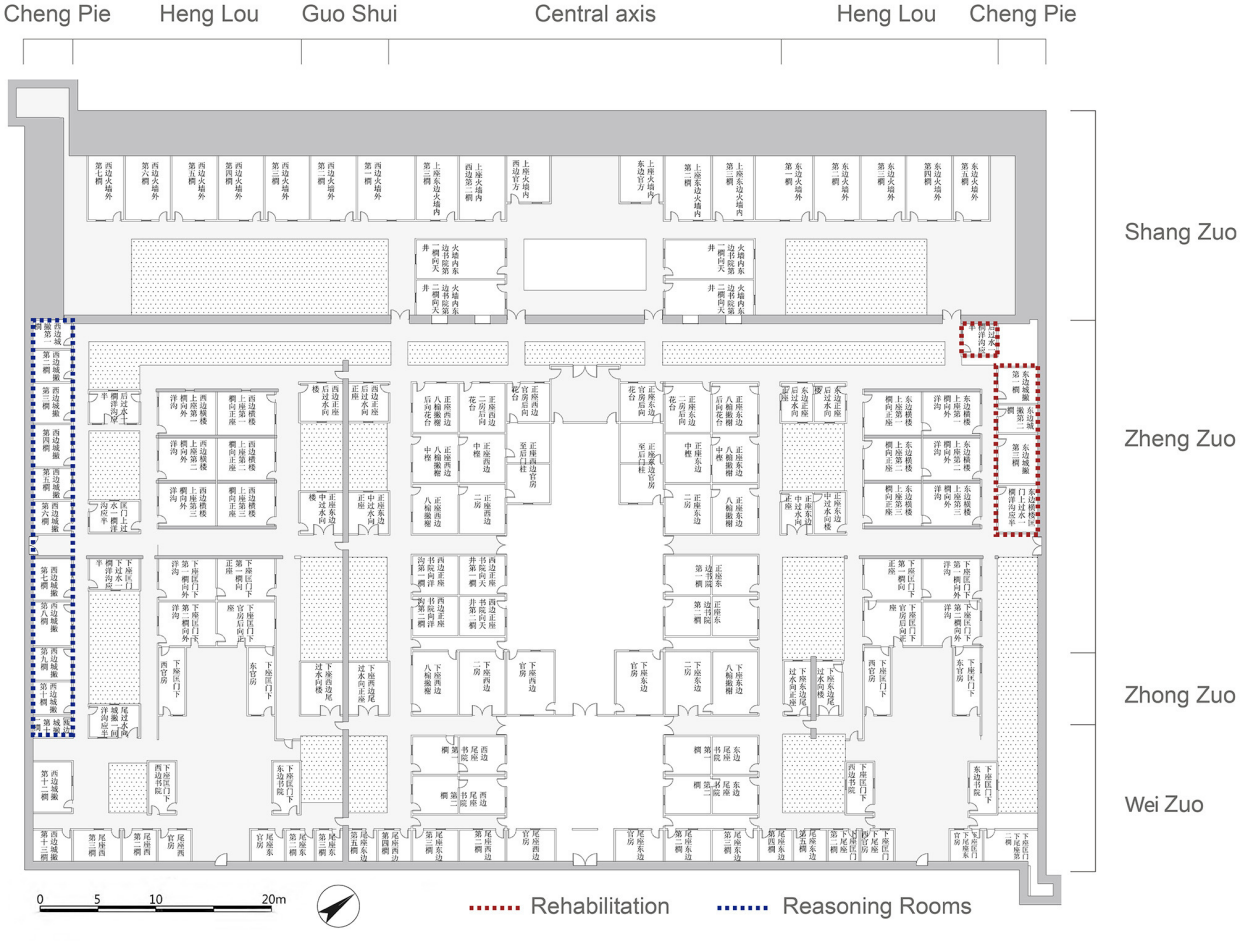
Detail of plan.

The Renhe Village data used in the study are mainly derived from the “Jiu Book” (Figure 3) and a field survey. The Jiu Book is a form of agreement for family division. It sets out the results of the brothers' negotiations on family property division (55). The “Jiu” in the Jiu Book is a number sheet recording the name of the property. The name “Jiu” is derived from the ancient practice of divination using a turtle shell.^11^ “Nian Jiu” (lot drawing) is an original distribution method intended to ensure fairness. A few small pieces of paper were used to write words or marks and were made into paper balls. Each person involved took one ball to determine the final distribution of the family property (56). The division is generally determined by “Nian Jiu,” which, to a certain extent, ensures the equity of the division. The Nian Jiu rule eliminates the doubts of sons about possible cheating in the family property division process. It ensures procedural justice in the division of family property. However, Nian Jiu is not used for random lot drawing for the whole building. It is used for each area to avoid the random domination of an area by one household branch, leading to an unfair overall distribution. It can also ensure that the space of each household branch intersects with the other spaces. According to the Jiu Book of Renhe Village, at the time of family division, the property was equally distributed among the three sons. There were three Jiu Books for Zhi, Ren, and Yong, each of which was marked, signed, and lots were cast. Then, each son kept a copy. At present, only the Jiu Book of Ren is left. It is the most important data source for the present research. The auspicious characters Zhi (wisdom), Ren (benevolence), and Yong (bravery) were used to represent the name of the household branch when dividing the family property, indicating that the elders in the family had good expectations for the three brothers. The eldest son's household branch is called Zhi Rooms, the second son's is Ren Rooms, and the third son's is Yong Rooms. Some houses were damaged, and there was no floor plan in the Jiu Book to determine room positioning. Therefore, this study carried out several field explorations from December 2021 to August 2022 and compared the literature of relevant cases and the Jiu Book of Renhe Village to determine the spatial location of the rooms and draw a building plan (Figure 4).

### 2.5. Methods

Based on the literature analysis, the overall pattern of the early establishment of Renhe Village was restored through the study of the writing method of the Jiu Book and interviews with descendants living in Renhe Village. The narration in the Jiu Book strictly follows the order from top to bottom and from the middle to both sides; there is a strict hierarchical orientation order, thereby forming a complete spatial positioning system. The space layout of Renhe Village from top to bottom is Shang Zuo, Zheng Zuo, Zhong Zuo, and Wei Zuo (Figure 4). The main function of Shang Zuo is as a school space, including an academy and bedrooms for women. The functions of the central part of Zheng Zuo and Wei Zuo include ritual functions and living functions. The overall structure includes the hall, bedrooms, and kitchen space. Wei Zuo is the area closest to the main entrance. It is used for certain ritual functions and living functions. The space includes the hall, bedrooms, and the academy. The Renhe Village building has complete internal functions and cross-distribution, thereby meeting the needs of life at that time.

The inferred restoration positioning of the damaged building space was established (Figure 4) through a field survey, data collection, and interview research. Due to the disrepair of many years, some rooms recorded in the Jiu Book of Renhe Village could not be found inside the building. The existing structure of Chen Pie^12^ in the west was rebuilt by descendants. According to Zhang Zuhuang, an eighth-generation descendant of the founder of Renhe Village, the pattern after the reconstruction deviates from the original pattern.

#### 2.5.1. Environmental simulation

The lighting and ventilation attributes of architectural physics were studied, and the correlation between the physical environment of the settlement and the characteristics of the settlement space was analyzed. For lighting analysis, at a point in the indoor reference plan, the ratio of the illuminance generated by directly or indirectly receiving diffuse light from the assumed and known sky brightness to the diffuse light generated simultaneously by the sky on the outdoor unobstructed horizontal plan was obtained. The average lighting coefficient was calculated according to a single room, namely, the arithmetic mean value of the lighting coefficient at each intersection point on the grid in the room. Computer simulation software was used to calculate and analyze the lighting quality and condition of each functional room.

The lighting coefficient C of a certain point in the room can be calculated as follows:


$$\begin{array}{l} C=\frac{E_{n}}{E_{w}}\times 100\%, \end{array}$$

where E_n_ represents indoor illumination, lx;

and E_w_ represents outdoor illumination, lx.

The analysis of room ventilation was performed using EcotectAnalysis software. The simulation location was set as Yongtai county, Fujian province, and the given wind speed and direction values were set according to the average values provided by the Bureau of Meteorology for each season of the year, namely, northwest wind in winter and southeast wind in summer. The Computational Fluid Dynamics method was used to establish the wind field, whereby the mathematical control equation of mass conservation, momentum conservation, and energy conservation of fluid flow was established in the analyzed calculation domain. Its form is as follows:


$$\begin{array}{l} \frac{\partial \left(\text{p}\varphi \right)}{\partial t}+div\left(p\overset{\cdot }{U}\varphi \right)=div\left(\Gamma_{\varphi }gra\varphi \right)+S_{\varphi } \end{array}$$

In this equation, ϕ can be physical quantities, such as speed, kinetic energy, turbulent dissipation, and temperature.

#### 2.5.2. Spatial syntax

Spatial syntax theory was applied to analyze and visually express the spatial features of Renhe Village, providing a basis for the analysis of spatial equity (18). Spatial syntax analysis, based on spatial topology, from the quantitative interpretation of geometric relations, can quantitatively analyze and visualize the spatial accessibility of body behavior and visual sense through visibility graph analysis (VGA). Based on the restored Renhe Village, a spatial analysis model was established. In the VGA model, based on the width of a person's normal step (about 700 mm), the space was set as a grid with n visibility units. Each grid element a_n_ can be understood as a person's visibility point. The space was remapped around this point, and the topological depth value of the central space, TotalDepth (a_n_), could be calculated. The accessibility among the points in the body and sightline indicates that the social and cognitive significance contained after the subdivision of convex space, namely any two potentially occupied positions are visible to each other and have potential social interaction. In the VGA model, each visibility point was generated and measured; the visible relationship between each point and other points was analyzed; and the accessibility measurement according to the sightline integration of different points was carried out. The formula is as follows:


$$\begin{array}{l} \text{Integration}\left(a_{n}\right)=\frac{\left[n\left(\log_{2}\left(\frac{n}{3}\right)-1\right)+1\right]\left(\frac{n}{2}-1\right)}{\left[\frac{\left(n-1\right)\left(n-2\right)}{3}\right]\left(\frac{\text{TotalDepth}\left({\text{a}}_{\text{n}}\right)}{n\text{-1}}-1\right)} \end{array}$$

The sightline integration measured the potential of the space attraction. It is a function of the reciprocal of the topological depth value. The higher the function value, the lower the global topological depth. It was divided into 10 levels from high to low. The sightline integration values of all elements were displayed in the corresponding colors to generate a global sightline integration analysis chart. Statistics and comparisons were made on the sightline integration values of each space obtained from the analysis of the visibility model under multiple scenarios. Then, the internal logic leading to the difference in its spatial fabric could be analyzed.

## 3. Analysis results

### 3.1. Natural unit indicator

**Figure 5:**
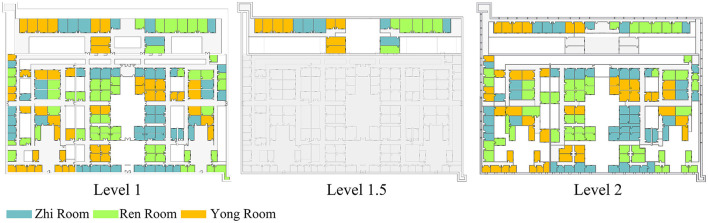
Distribution of the household branch.

The analysis of the natural unit indicator is based on room units in the building. The study was conducted from the perspective of three evaluation indices: room quantity, lighting, and ventilation. There is a difference between the used plan and the actual height of the building. The building itself has a terraced layout, meaning that the building plan can be positioned according to the usage habits of the ancient people, and the part with a small height difference, namely the actually used coherent space, was defaulted to the same horizontal plan; therefore, the final plan has three levels: 1, 1.5, and 2. The number of rooms and space location of each household branch were determined, and the plan of the divided rooms could be determined (Figure 5) through the interpretation of the information in the Jiu Book and the space positioning method. When analyzing the lighting and ventilation values, the building plan poses the problem of platform elevation difference. To reduce shielding, Wei Zuo and Zheng Zuo were connected and adjusted through model correction. Different elevations were adopted as the benchmark to adjust the model plan. The three elevations determined are the cross-sections of the actual altitude. Therefore, the lighting and ventilation analysis could be more accurate.

#### 3.1.1. Regional rooms

The room quantity statistics can be summarized as follows: In Table 1, based on the number of levels, the overall building was divided into two major areas. Area 1 comprises four large blocks, namely Zheng Zuo, Zhong Zuo, Shang Zuo, and Wei Zuo. Area 2 comprises East Side, West Side, and Kuang Door.^13^ The number of rooms in Area 1 in proportion to the total number of rooms was calculated, and the data are as follows:

**Table 1:** Statistics of rooms.

**Floor**	**Area 1**	**Area 2**	**Zhi rooms**	**Ren rooms**	**Yong rooms**	**Public room**	**Total**
Level 1	Zheng Zuo	East Side	12^*^	8^#^	10	/	30
		West Side	7^#^	12^*^	9	/	28
		Kuang Door	1^*^	0^#^	0^#^	/	1
		Sum	20^*^	20^*^	19	/	59
		Proportion	18.69%	19.05%^*^	18.63%	0.00%	19.28%
	Zhong Zuo	East Side	8^*^	1^#^	3	/	12
		West Side	5	11^*^	2^#^	/	18
		Kuang Door	2^#^	2^#^	5^*^	/	9
		Sum	15^*^	14	10^#^	/	39
		Proportion	14.02%^*^	13.33%	9.80%^#^	0.00%	12.75%
	Shang Zuo	Firewall Interior	2	2	2	/	6
		Firewall External	5^#^	5^#^	6^*^	/	16
		Sum	7^#^	7^#^	8^*^	/	22
		Proportion	6.54%^#^	6.67%	7.84%^*^	0.00%	7.19%
	Wei Zuo	East Side	4	6^*^	3^#^	1	14
		West Side	4	3^#^	8^*^	1	16
		Sum	8^#^	9	11^*^	2	30
		Proportion	7.48%^#^	8.57%	10.78%^*^	0.66%	9.80%
	Total		50^*^	50^*^	48^#^	2	150
	Proportion		46.73%^#^	47.62%^*^	47.06%	0.66%	49.02%
Level 1.5	Shang Zuo	Firewall Interior	2^#^	2^#^	4^*^	/	8
		Firewall External	4	4	4	/	12
	Total		6^#^	6^#^	8^*^	/	20
	Proportion		5.61%^#^	5.71%	7.84%^*^	0.00%	7.19%
Level 2	Zheng Zuo	East Side	12^*^	8^#^	10	/	30
		West Side	7^#^	12^*^	9	/	28
		Kuang Door	1^*^	0^#^	0^#^	/	1
		Sum	20^*^	20^*^	19^#^	/	59
		Proportion	18.69%	19.05%^*^	18.63%^#^	0.00%	19.28%
	Zhong Zuo	East Side	8^*^	1^#^	3	/	12
		West Side	5	11^*^	2^#^	/	18
		Kuang Door	2^#^	2^#^	5^*^	/	9
		Sum	15^*^	14	10^#^	/	39
		Proportion	14.02%^*^	13.33%	9.80%^#^	0.00%	12.75%
	Shang Zuo	Firewall Interior	2	2	2	/	12
		Firewall External	4	4	4	/	6
		Sum	6	6	6	/	18
		Proportion	5.61%^#^	5.71%	5.88%^*^	0.00%	5.88%
	Wei Zuo	East Side	5	6^*^	3^#^	/	14
		West Side	5	3^#^	8^*^	/	16
		Sum	10	9^#^	11^*^	/	30
		Proportion	9.26%	8.49%^#^	10.78%^*^	0.00%	9.80%
	Total		51^*^	49	46^#^	/	146
	Proportion		47.66%^*^	46.67%	45.10%^#^	0.00%	47.71%

* The best evaluation index.

# The worst evaluation index.

#### 3.1.2. Physical environment

**Figure 6:**
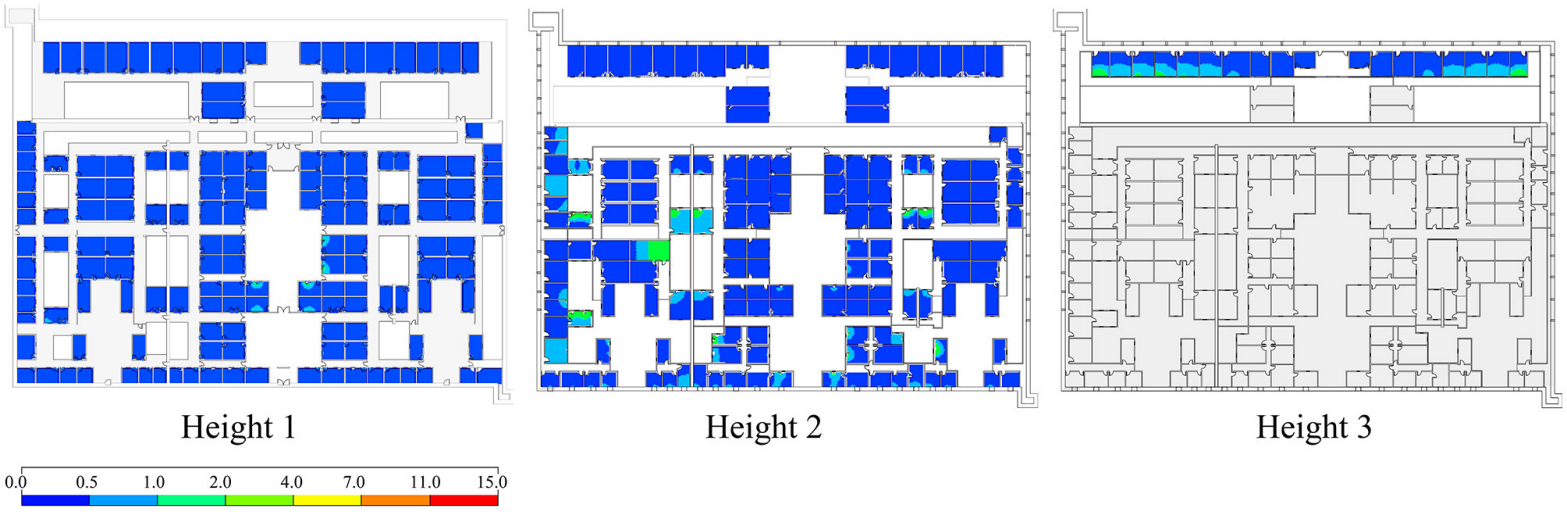
Analysis of lighting.

**Figure 7:**
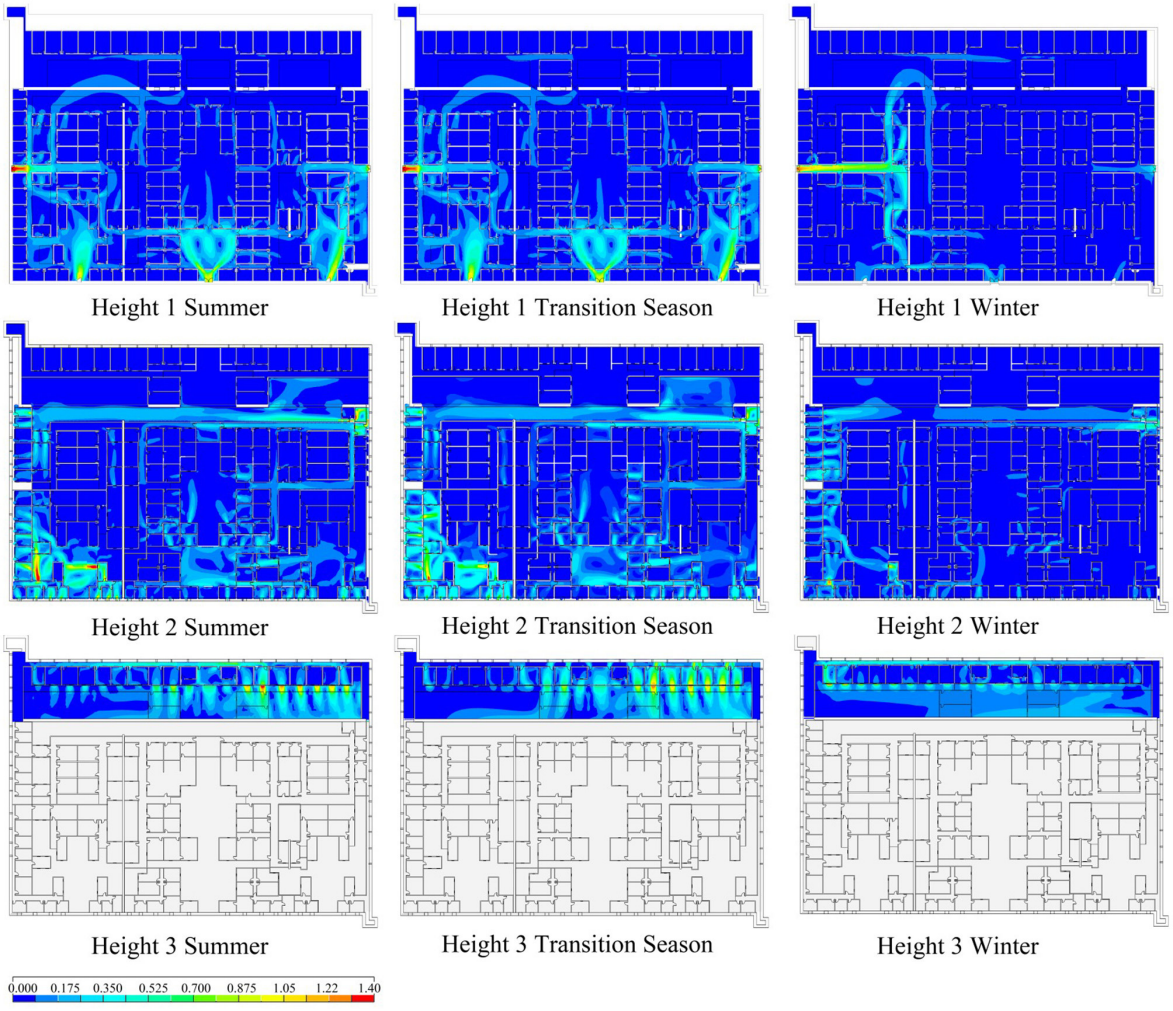
Analysis of ventilation.

**Figure 8:**
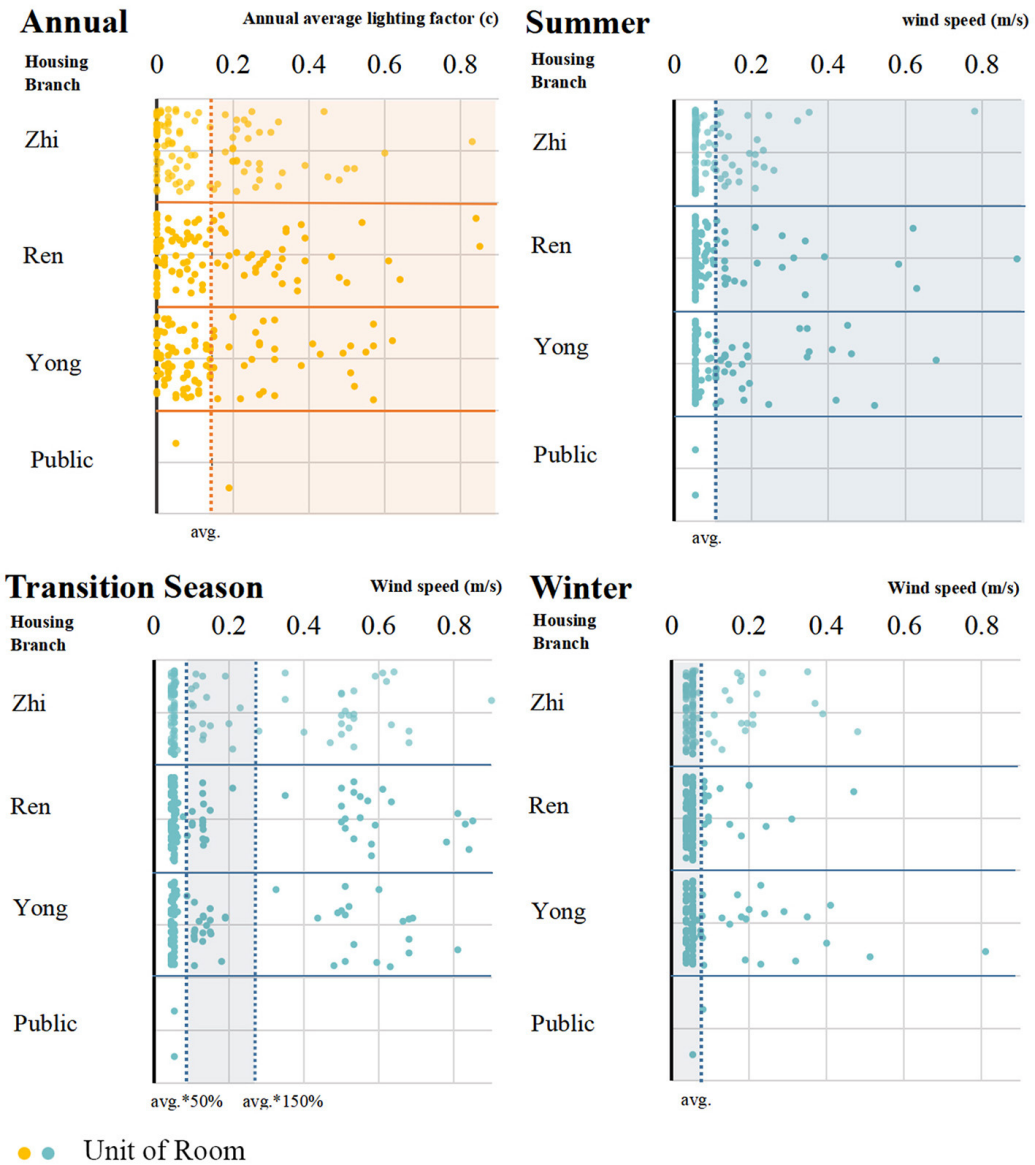
Statistical chart of lighting and ventilation.

The corresponding lighting and ventilation values of each room were calculated (Figures 6–8), and the average of the lighting and ventilation values of all rooms were taken as the relative measurement standard of the building (Table 2). Traditional Chinese residential buildings contain the wisdom of people of all ages. The development of residential buildings in the late Qing Dynasty reached its peak, and the overall technical area was complete. Therefore, the architecture and life of the village adapted to each other, and the ventilation and lighting of the building were acceptable to the people at that time. However, the standards differ from modern architectural design standards and cannot be measured according to current specifications. Therefore, the average value of ventilation and lighting in Renhe Village was taken as the relative standard in the overall proportion. The indoor lighting of Renhe Village comes mainly from the patio. There are small windows or even no windows on the high walls of the rooms, resulting in a poor indoor lighting environment. The lighting value of Renhe Village is far lower than the standard lighting value of modern residential buildings. As a result, in the case of poor overall lighting, the average value was selected as a relatively fair standard to judge the rooms with excellent lighting. The average lighting value of Renhe Village is 0.145. If the lighting value is higher than the average value, it refers to a room with relatively good lighting, and vice versa. Similarly, the ventilation is consistent with the lighting, and the average ventilation value of the corresponding season is used as the standard. In summer, residential buildings should focus on ventilation. If the ventilation value is greater than the average value of 0.107 m/s, it should be a relatively comfortable room. In the transition seasons (Spring and Autumn), the average value of 0.182 m/s, fluctuating by 25%, is used as the standard. Within the range, the ventilation is relatively comfortable. However, in winter, people need to keep warm. If the ventilation value is less than the average value of 0.084 m/s, it would be comfortable. The calculation results are shown in Figure 8.

**Table 2:** Statistics of lighting and ventilation.

**Indicators**	**Season**	**Zhi rooms**	**Ren rooms**	**Yong rooms**	**Public room**	**Average**
Lighting factor (c)	Year-round	0.122	0.169	0.143	0.120	0.145
Wind speed (m/s)	Summer	0.096	0.111	0.115	0.055	0.107
	Transition season	0.187	0.188	0.173	0.055	0.182
	Winter	0.080	0.081	0.090	0.068	0.084

The natural unit indicator of the building was finally obtained using the above method, which is the number of rooms on each floor corresponding to each household branch, and the number of rooms with good lighting and ventilation quality (Table 3).

**Table 3:** Statistics of nature unit indicator.

	**Zhi rooms**	**Ren rooms**	**Yong rooms**	**Public room**	**Total**
Quantity	107^*^	105	102^#^	2	316
Lighting-comfortable	37	39^*^	31^#^	1	108
Ventilation-comfortable	43	42^#^	46^*^	1	129

* The best evaluation index.

# The worst evaluation index.

### 3.2. Overall spatial indicator

**Figure 9:**
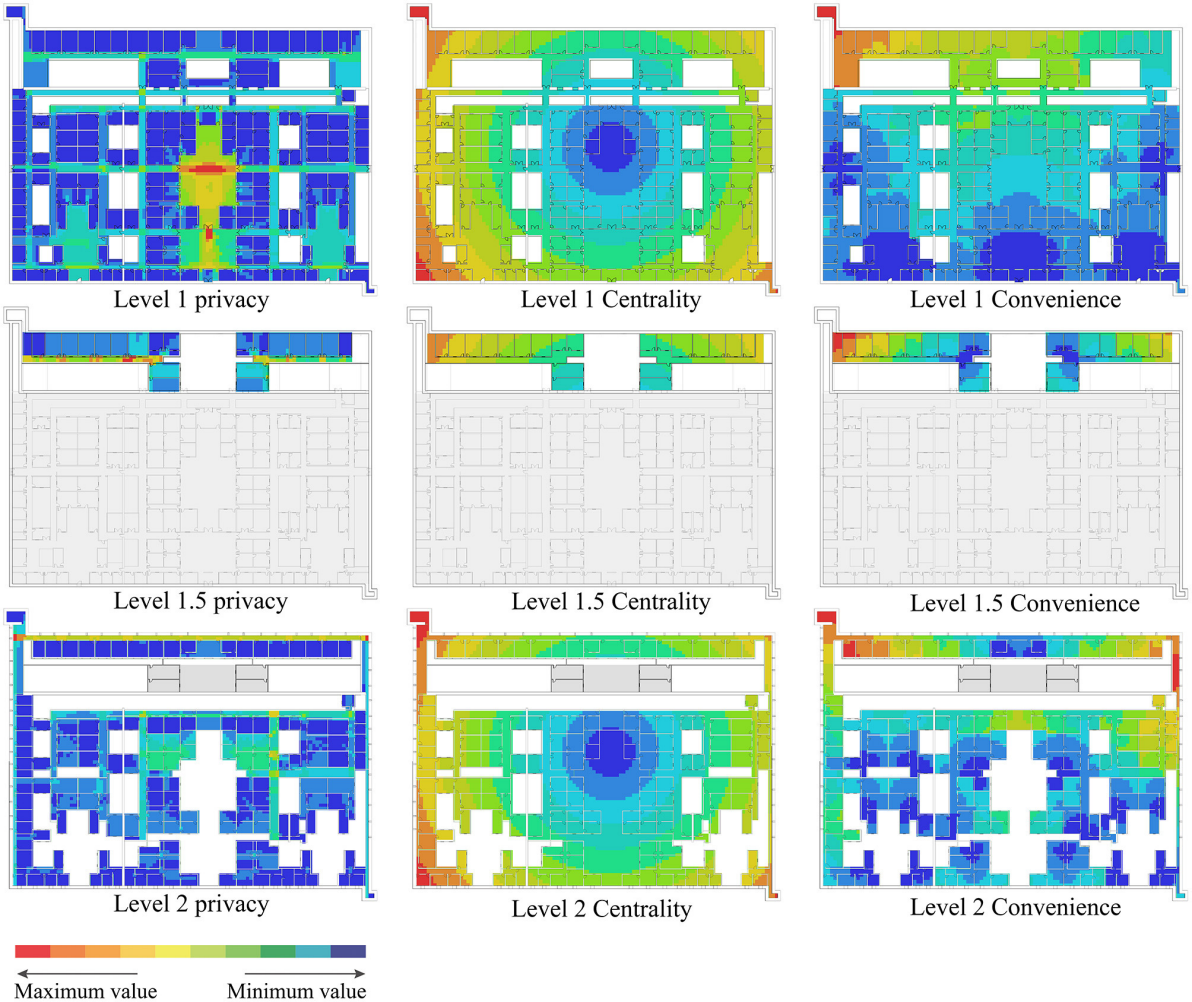
Overall spatial indicator analysis chart.

The overall spatial indicator is generated by a review based on the privacy, centrality, and convenience of different housing spaces. The gradient from blue to red shows the values of the three evaluation indices. The redder the color, the greater the value, and vice versa. The greater the privacy value of the rooms inside the building, the higher the spatial connectivity, and the lower the degree of privacy. The greater the value of centrality, the weaker the centrality. For the indicator of convenience, the greater the value, the weaker the convenience of the room. The overall value of room privacy, centrality, and convenience was divided by the number of rooms in the corresponding household branch, and finally, the average value was taken as the overall indicator to measure the space. Figure 9 and Table 4 show the results of the analysis.

**Table 4:** Statistics of overall spatial indicator.

	**Zhi rooms**	**Ren rooms**	**Yong rooms**	**Public room**	**Total**
Privacy	24.728^*^	29.547^#^	28.073	13.453	27.338
Centrality	29.405	25.101^*^	32.377^#^	31.769	28.949
Convenience	30.163^#^	28.194	27.389^*^	10.761	28.491

* The best evaluation index.

# The worst evaluation index.

## 4. Discussion and conclusion

### 4.1. Discussion

#### 4.1.1. Natural unit indicator

First, according to the allocated number of rooms for each household branch, the equality idea of “reducing more to replenish less” is verified. The power order of traditional residential buildings has a linear correlation with the spatial layout, and the spatial layout hierarchical order decreases from the middle to both sides, from the inside to the outside, from the north to the south, and from the east to the west (57). Renhe Village is the central part of the traditional residential buildings and enjoys the highest status. Area 1 follows the hierarchical order of “Zheng Zuo > Zhong Zuo > Shang Zuo > Wei Zuo,” and Area 2 follows the hierarchical order of “East > West > Kuang Door” and “Interior Firewall > External Firewall.” Based on the number of levels, Table 1 shows the statistics on the number of room areas by subdividing Area 1 and Area 2. Since the number of rooms in each household branch is different, the proportion of corresponding rooms in the total number of rooms of the household branch was selected as the criterion to judge the proportion of the number of rooms in the total number of individuals. The comparison of the total number of rooms at the first level shows that the proportion of the number of rooms in Ren Rooms at the first level to the total number of rooms is 47.62%, greater than that of the other two brothers' rooms. The proportion of the Yong Rooms at Level 1.5 is 7.84%, ranking first, and the proportion of the Zhi Rooms at Level 2 ranks first. Therefore, each of the three brothers occupies an advantage at one of the levels, meaning that the proportions are relatively balanced. In Area 1 on the second level, on the first and second floors, the number of rooms in Zhi Rooms and Ren Rooms in Zheng Zuo is 20, but Ren Rooms has the highest proportion of rooms among the three. The data in Zhong Zuo show that the number of rooms in the first and second levels of Zhi Rooms is 14.02%, the highest among the three. The data on Shang Zuo and Wei Zuo indicate that the total number and proportion of rooms in Yong Rooms is the highest among the three. In Zheng Zuo and Zhong Zuo, at higher levels, Zhi Rooms and Ren Rooms each occupy one, while Shang Zuo and Wei Zuo are spaces with lower levels, and Yong Rooms are compensated in quantity, thereby achieving a balance among the three. The data on Area 2 at the third level show that, in the whole building, the optimal number of rooms in the three household branches accounts for six areas, respectively. For example, there are 12 rooms in the east of Zheng Zuo on the first and second levels, one in Kuang Door, and eight in the east of Zhong Zuo. These six areas all have the largest number of rooms in the corresponding areas of the corresponding levels. There are 12 rooms in the west of Zheng Zuo at the first and second levels of Ren Rooms, 11 rooms in the west of Zhong Zuo, and six rooms in the east of Wei Zuo. These three areas have the largest number of rooms in the corresponding six areas of the corresponding levels. The three areas at the first level of Yong Rooms, namely Zhong Zuo, Shang Zuo, and Wei Zuo, correspond to Kuang Door, External Firewall, and the West. Yong Rooms has the largest number of areas in the Interior Firewall at Level 1.5 in Shang Zuo, which also has the largest number of rooms in Kuang Door at Zhong Zuo and West at Wei Zuo on the second level. Yong Rooms has the largest number of the six areas in total. The result shows that, in the process of room distribution, the number of rooms in one part of the area is large, and the number of rooms in the other part is correspondingly small, and vice versa, ultimately reflecting the fair distribution method of “reducing more to replenish less”.

Second, the allocation of rooms among the household branches fully demonstrates the principle of “intersection and interpenetration”. As the room distribution plan (Figure 4) and the number of areas (Table 1) show, in terms of the number of rooms Zheng Zuo, Zhong Zuo, Shang Zuo, and Wei Zuo, the three branches account for part of each, and no single space is dominated by one branch. In addition, the proportion of rooms is relatively equal, and the rooms in each area are fairly distributed. For example, there are 12 rooms in the east of Zheng Zuo for Zhi Rooms on the first level, eight for Ren Rooms, and 10 for Yong Rooms. The average number of rooms in this area is 10. In the west, there are seven for Zhi Rooms, 12 for Ren Rooms, and nine for Yong Rooms, an average of eight in this area. Near Kuang Door, there is one room for Zhi Rooms, zero for Ren Rooms, and zero for Yong Rooms. In the final statistics, there are 20 rooms for Zhi Rooms and Ren Rooms, and 19 for Yong Rooms. On the whole, the number of rooms for each is practically the same. The same is true for other areas. Irrespective of the number of single-area spaces or the overall areas in Zheng Zuo, the quantity of rooms is relatively equal without a significant difference. Each area penetrates into the other, and each household branch has rooms in each area of the building, thereby ensuring the overall equity of the physical attributes of lighting and ventilation in the rooms of each household branch.

In short, in terms of the natural unit indicator, the equity of housing property distribution in Renhe Village takes into account not only the number of rooms but also the number of rooms with reasonable physical attributes of lighting and ventilation. The results of the Renhe Village room distribution (Table 3) showed that the number of rooms for Zhi Rooms is 107, the number of rooms with good lighting quality in Ren Rooms is 39, and the total number of rooms with good ventilation quality in Yong Rooms is 42, all of which are the optimal values of the corresponding attributes. Hence, each of the three household branches has the best proportion in the corresponding overall number of rooms, and the number of rooms with good lighting and ventilation, thereby achieving balance in the equity of the natural unit indicator among the three household branches.

#### 4.1.2. Overall spatial indicator

Data simulation analysis revealed that equity is shown in the distribution of housing property rights in Renhe Village in the overall spatial indicator (privacy, centrality, and convenience). As Table 4 shows, the privacy value refers to the average distance of the visible area of each room. A smaller privacy value means that the room can be viewed in a small range, indicating a higher degree of privacy. The privacy value of Zhi Rooms is 224.728 m, the lowest among the three rooms; therefore, it has the highest degree of privacy. The centrality value of Ren Rooms is 25.101 m, meaning that the average distance from Ren Rooms to the center is the shortest and the centrality is the strongest. The convenience value of Yong Rooms is 27.389 m, smaller than the 30.163 m of Zhi Rooms and the 28.194 m of Ren Rooms, indicating that the distance to the entrance and exit is the shortest, making it most convenient for Yong Rooms. In short, when allocating the rooms, each of the three household branches occupies an optimal value for the three evaluation indices of the overall spatial indicator, and the allocation is very balanced. Therefore, it can be inferred that, in terms of the overall spatial indicator, the distribution of property rights in Renhe Village is equitable.

#### 4.1.3. Equity evaluation model

The model equation for evaluating the equity of housing property distribution was based on the above discussion of the results for equity. The study established a computational model for quantifying fairness by branching qualitative overall existence and local fairness. The calculation method used the specific values of different branch attributes of each house faction to establish the matrix equation. The joint cubic equation to find the proportion of each coefficient can be obtained from one set of coefficient solutions. The model was based on the natural unit indicator and the overall spatial indicator, and the final balance coefficient was obtained according to the research data.

(1) Natural unit indicator (Par): The unit indicator value of equity that represents the number of rooms in an individual building space, the number of rooms with good lighting quality, and the number of rooms with good ventilation. The model can be expressed as Formula (1):


(1) $$\begin{array}{l} Par=\beta_{1}Qua+\beta_{2}Day+\beta_{3}Win, \end{array}$$

where Qua represents the number of rooms, Day represents the number of rooms with good lighting quality, and Win represents the number of rooms with good ventilation. Their units are constant n. Renhe Village is taken as representative, and β_1_:β_2_:β_3_≅ 0:1:2.

During the construction and distribution of traditional Chinese residential buildings, attention was paid to the quality of room ventilation. However, the structural trend is characterized by a scarcity of ventilation and lighting resources. During the division of property, one room with good ventilation is equivalent to two rooms with good lighting. Renhe Village is a building with a patio. The 1:2 distribution ratio is defined based on the ventilation and lighting characteristics of the architectural structure of Renhe Village and can be applied to architectural spaces of the same type. Therefore, it can be further understood that the ancients did not require a high degree of lighting but regarded ventilated rooms as a more scarce and comfortable space.

(2) Overall spatial indicator (Int): Based on the average spatial distance of the room in each household branch, the relationship between the privacy, centrality, and convenience of the rooms and the overall building was studied. The model can be expressed as Formula (2):


(2) $$\begin{array}{l} Int=\alpha_{1}Pri+\alpha_{2}Cen+\alpha_{3}Con, \end{array}$$

where Pri is privacy, Cen represents centrality value, Con represents convenience value, and their units are distance in meters. Renhe Village is taken as representative, and α_1_:α_2_:α_3_ ≅ 2.62:1:4.23.

The ancients placed the most value on room centrality, followed by privacy and convenience. They also paid more attention to the traditional ritual hierarchy than anything else. Renhe Village is a space where a large family lived together; therefore, the privacy aspect was a minor consideration. Moreover, diligence and simplicity are typical characteristics of traditional Chinese farmers. Therefore, their requirements for convenience were low.

Equity is judged according to the direct comparison among brothers. *Par*_1_ *and Par*_2_ are used for the natural unit indicator. The overall spatial indicator of two brothers is represented by *Int*_1_and *Int*_2_. The more the final absolute value of equity approaches 1, the more equitable the distribution is between the two brothers. Its model can be expressed as Formula (3):


(3) $$\begin{array}{l} \frac{\left|Par_{1}\right|}{\left|Par_{2}\right|}\to 1\frac{\left|Int_{1}\right|}{\left|Int_{2}\right|}\to 1 \end{array}$$

With the increase in the number of participants in the family property division, the equity of the natural unit indicator becomes *Par*_1_, *Par*_2_, *Par*_3_, *Par*_4_, *Par*_5_, *Par*_6_, etc., accordingly, and the demonstration parameters of the equity of the overall spatial indicator can be increased to *Int*_1_, *Int*_2_, *Int*_3_, *Int*_4_, *Int*_5_, *Int*_6_ etc. Therefore, regardless of the number of research objects, the formula can provide a certain test standard of equity for building allocation.


(4) $$\begin{array}{l} \frac{\left|\text{In}{\text{t}}_{1}\right|}{\left|\text{In}{\text{t}}_{2}\right|}\to \frac{\left|\text{In}{\text{t}}_{2}\right|}{\left|\text{In}{\text{t}}_{3}\right|}......\frac{\left|\text{In}{\text{t}}_{\text{n}}\right|}{\left|\text{In}{\text{t}}_{\text{n}+1}\right|}\to 1\;\\ \frac{\left|\text{Pa}{\text{r}}_{1}\right|}{\left|\text{Pa}{\text{r}}_{2}\right|}\to \frac{\left|\text{Pa}{\text{r}}_{2}\right|}{\left|\text{Pa}{\text{r}}_{3}\right|}......\frac{\left|\text{Pa}{\text{r}}_{\text{n}}\right|}{\left|\text{Pa}{\text{r}}_{\text{n}+1}\right|}\to 1 \end{array}$$

Taking Renhe Village as an example, under the guidance of formulas, when the houses are redistributed with functional property rights, the newly divided property rights should meet the establishment of Formula (4), and the equitable distribution of the house property can be reasonably calibrated. The equity formula in the Renhe Village model can also be used as a reference for modern housing distribution.

### 4.2. Implications

#### 4.2.1. Traditional residences

Through accurate quantitative analysis, this study verified the existence of objective equity in Chinese traditional Zhuangzhai from the two levels of natural unit indicator and overall spatial indicator. Based on this, an equity model is derived. The model can be used to identify and test equity when Renhe Village is divided again. In addition, the coefficient in the formulas of Renhe Village can be used as a reference to test the distribution rationality of other villages, providing a quantitative weight standard for the distribution equity of traditional residential buildings and giving corresponding inspiration to other Zhuangzhai villages to be divided.

#### 4.2.2. Contemporary residences

In modern China, rural housing distribution lacks connection and inclusiveness, which can lead to a lack of cultural integration. Therefore, the results of this study can help us recognize the meaning behind cultural phenomena, reconnect bloodlines, and promote the development of family inheritance culture. Compared with the traditional division method of “combination in division” of Renhe Village, modern rural housing distribution in China involves simple division and independent living. In modern China, when parents in rural areas distribute their property, they usually give each brother one floor. If the houses are in a row, each brother gets one house and there is no connection between the houses. There is no inclusive relationship of mutual integration, which is not conducive to the common development of brothers. Therefore, arising from the present research on the family division of Renhe Village under traditional culture, suggestions on equity can be given for modern rural housing distribution. This could restore the influence of traditional culture on the family and promote family development.

The results of this study have significant reference value for reviewing the equity of property rights distribution of social security housing in modern cities. With the improvement of living standards and the global policy of combining rent and sale, rent sharing has become part of the lifestyle of modern people. In real life, social security housing has many problems, such as uneven living conditions and unbalanced distribution under the condition of equal rent, sale, or rental prices, resulting in a decline in overall residential satisfaction. Therefore, taking the improvement of the current situation of public rental apartments as a starting point, the research results of this study reflect the equity attributes of modern apartments. The discussion on the design strategy of the equity of shared apartments ensures the equity of the property distribution rights of social security housing, thereby providing a reference for the public humanistic health system in the modern living environment.

### 4.3. Conclusion

Based on the theory of “equal share for all sons” in ancient China and the “equity” and “justice” that are of modern significance, this study analyzed the family division culture of individual traditional housing and the corresponding impact indices of family division equity. The main results were as follows: Taking Renhe Village as the research object, this study built a spatial syntax data model and 3D simulation technology for the simulation analysis of space and climate. The results showed that Renhe Village meets the requirements of the equity evaluation system of housing property rights distribution in terms of the natural unit indicator (quantity, lighting, ventilation) and the overall spatial indicator (privacy, centrality, convenience). In other words, equity does not mean an absolute average share, but an equity culture formed after six evaluation indices under the subdivision of two indicators are balanced. The proposed equity system is an important way of improving living conditions. This study applied both qualitative and quantitative research methods, thereby enriching the research dimension of equity in the division of traditional Chinese residential buildings. Meanwhile, the research results can provide an equity evaluation criterion for other Zhuangzhai buildings, including Renhe Village, as well as a quantifiable criterion for the distribution of modern rural housing and social security settlements. These achievements will be conducive to the development of a humane health culture, and ultimately promote the protection, inheritance, and rational utilization of the village.

### 4.4. Limitations and implications

Although the study has conducted an in-depth discussion on the natural unit indicator and the overall spatial indicator of Renhe Village in combination with a field survey and historical data, the following limitations exist: (1) Renhe Village has a long history, and some building structures have been damaged and cannot be restored. The restoration of buildings was conducted based on the rationalization inference of the site status and interviews with the descendants of the ancients of Renhe Village. Therefore, there may be minor errors in the research data. (2) The original room functions of Renhe Village cannot be verified, resulting in the lack of practical and verifiable data on the use of space and a lack of further evidence in measuring the equity attributes. (3) Due to the damage to the building, the temperature, and the noise at the site, the true value of the original space cannot be accurately restored. Therefore, the environmental data studied are mainly the statistics of lighting and ventilation. These data do not combine the noise, comfort, and temperature measured on-site (36, 37) to evaluate the equity in terms of natural unit indicators. The result is that there are deficiencies in the equity evaluation model of housing property rights distribution. (4) This study applied spatial syntax in a case study of the spatial form of Renhe Village, without a comprehensive comparative study of multiple objects. The conclusions still need to be verified in future research and practical application.

In conclusion, equity is always the core value concept rooted in the hearts of all people, and humanistic public health involves the construction of a healthy natural and cultural environment for human society. In recent years, research on Zhuangzhai buildings in Yongtai has involved qualitative research on the expectations of cultural value, spatial form, and detailed decoration. However, up to now, research on how to interpret the humanistic health of Zhuangzhai architecture and how to interpret Zhuangzhai culture using scientific data and applying this to modern society has not been undertaken.

## Data availability statement

The datasets generated or analyzed during the current study are not publicly available due to the privacy of others but are available from the corresponding author on reasonable request. Requests to access these datasets should be directed to Kaidachenwhu@gmail.com.

## Author contributions

KC and MS contributed to conception and design of the study. JL organized the database. KC and SD performed the statistical analysis. MS wrote the first draft of the manuscript. KC, JL, YW, and SD wrote sections of the manuscript. All authors contributed to manuscript revision, read, and approved the submitted version.

## Footnotes

^1^*The Sequel to the Biography of Yan Lun of Wu* is a collection of ancient Chinese myths and supernatural stories, written by Wu Jun in Liang State of the Southern Dynasty, and is a sequel to the biography of Yan Lun of Wu State.

^2^The property was reduced for people who get more to replenish people who get less to finally maintain balance.

^3^The spaces intersect and penetrate each other.

^4^Chinese ancient thinker, philosopher, writer, historian, and the founder and main representative of the Taoist school.

^5^The law of the movement and change of nature, principles of nature, will of nature, etc.

^6^House branch refers to the branch of a family. Each son has a small family, namely a house branch.

^7^To keep a certain form of the union after separation.

^8^The three-entrance courtyard means that there are courtyards in the courtyard. People can reach the large central courtyard after entering three courtyards. Generally, the central courtyard is owned by a rich and powerful family.

^9^The affection and psychology of mutual respect and mutual closeness between parents and children.

^10^The hall in the middle of the Zheng Zuo is the space with the highest order of ritual system in traditional Chinese residential buildings.

^11^Tortoise shells used for lot drawing.

^12^Cheng Pie refers to the room near the east and west perimeter walls.

^13^Door set on the firewall.
